# Air-Drying Llama Sperm Affects DNA Integrity

**DOI:** 10.3389/fvets.2020.597952

**Published:** 2020-12-10

**Authors:** María Ignacia Carretero, María Graciela Chaves, Claudia Cecilia Arraztoa, Fernanda Gabriela Fumuso, Mariana Carla Gambarotta, Deborah Margarita Neild

**Affiliations:** ^1^Facultad de Ciencias Veterinarias, Universidad de Buenos Aires, Buenos Aires, Argentina; ^2^Instituto de Investigación y Tecnología en Reproducción Animal, Universidad de Buenos Aires, Buenos Aires, Argentina; ^3^Consejo Nacional de Investigaciones Científicas y Técnicas (CONICET), Buenos Aires, Argentina

**Keywords:** air-dried, chromatin, DNA, llama, preservation, sperm

## Abstract

The objective of this study was to evaluate the effects of air-drying preservation on llama sperm DNA. Semen collections were carried out using electroejaculation under general anesthesia. A total of 16 ejaculates were processed from 4 males (*n* = 4, *r* = 4). Each sample was diluted 4:1 in a collagenase solution in TALP media, then incubated and centrifuged at 800 g for 8 min. The pellet was re-suspended to a concentration of 20 million sperm/ml in TALP. Then the samples were placed onto sterile slides forming lines and were left to dry under laminar flow for 15 min. After this, the slides were placed into Falcon centrifuge tubes and kept at 5°C. Sperm characteristics (motility, membrane function, viability and morphology) were evaluated in raw semen and in the air-dried samples kept at 5°C for 30 min. DNA evaluation (integrity and degree of chromatin condensation) was carried out in raw semen and in the air-dried samples after 30 min, 7, 14, 21, 30, and 60 days after preservation. To compare raw semen to the air-dried samples, a Wilcoxon test was used for all sperm characteristics except for DNA, where a paired Student *t*-test was applied. A split plot design was used to compare chromatin condensation between the different periods of preservation and a Kruskal Wallis test was used to compare DNA integrity. Motility, membrane function, viability and sperm with intact DNA decreased in the air-dried samples (*p* < 0.05), while morphology and chromatin condensation were not affected (*p* > 0.05). No significant differences were observed in the percentage of sperm with condensed chromatin between the different periods of preservation (*p* > 0.05). On the other hand, a significant decrease in the percentage of sperm with intact DNA was observed as from day 7 of preservation (*p* < 0.05). In conclusion the air-drying process has a negative effect on llama sperm DNA, hence the media used will need to be improved to protect DNA and be able to implement this technique in this species.

## Introduction

Artificial insemination (AI) with cryopreserved semen is a very useful tool for applying genetic improvement in herds; however, this biotechnology has not been effective in South American camelids (SACs). Attempts to extrapolate successful protocols in other species without considering the distinctive reproductive characteristics of SACs have led to errors and a slow development of AI in these species, obtaining pregnancy rates between 0 and 33% with cryopreserved semen (1–7).

Alternative preservation methods such as dehydration or air-drying sperm have been explored in different species [man: (8); mice: (9); and horses: (10, 11)]. These non-conventional techniques have the disadvantage that sperm become immotile and have impaired membranes, making it necessary to recur to intracytoplasmic sperm injection (ICSI) to obtain embryos. However, dehydration or air-drying have the advantages of easy preparation and low cost preservation of the samples, especially valuable for use in the field under difficult and often precarious conditions, as is the case of most of the work carried out in SACs and especially the wild species. In addition, with these methods the samples can be transported at 5°C, on slides or in tubes, without the need for cryoprotectants or liquid nitrogen and can also be used to conserve sperm recovered from the epididymis of post mortem individuals, allowing the conservation of genetic material of wild and/or endangered species.

Despite the various advantages of these alternative techniques, it is still unknown whether the sperm genetic material suffers any alterations over storage time. Alonso et al. (11) observed a higher percentage of DNA fragmentation in equine air-dried sperm compared to the control (cooled sperm), but the number of the samples was too low for a statistical analysis. Is interesting to highlight that sperm DNA quality is a very important parameter to evaluate because assisted reproductive techniques make it possible for sperm with damaged DNA to fertilize (12). Although the oocyte can repair a certain degree of DNA damage after fertilization, once this threshold is exceeded one or two effects are seen: (1) the percentage of embryo development decreases, known as embryo development block (13), and/or (2) early embryo loss is observed, also identified as a late paternal effect (14, 15). The ability to repair this damage would seem to be related to the genomic and cytoplasmic quality of the oocyte and to the amount of chromatin damage of the spermatozoa (13). Therefore, it is impossible to ignore the role of paternal DNA in early embryo development (16) and consequently, sperm DNA evaluation becomes important.

In this context, the objective of this study was to evaluate the effect of air-drying on llama sperm DNA.

## Materials and Methods

### Reagents

Collagenase, 6-carboxyfluorescein diacetate, dimethyl sulfoxide, propidium iodide, toluidine blue, agaroses and the reagents for the TALP medium were purchased from Sigma Chemicals (Sigma Aldrich, Buenos Aires, Argentina). Type I collagenase (*Clostridium peptidase A from Clostridium histolyticum*) was used. The TALP medium was prepared according to Parrish et al. (17): NaCl (114 mM), KCl (3.1 mM), NaHCO_3_ (2 mM), NaH_2_PO_4_ (0.3 mM), Na lactate (10 mM), CaCl_2_ (2 mM), MgCl_2_ (0.5 mM), Pyruvate (0.2 mM), HEPES (10 mM) and bovine serum albumin (3 mg ml/1).

### Animals and Location

The study was carried out at the Faculty of Veterinary Sciences of the University of Buenos Aires, in Buenos Aires, Argentina. The city is situated at sea level, latitude 34° 36′ and longitude 58° 26′.

For the study, 4 male *Lama glama* ranging between 7 and 11 years of age and weighing 140.75 ± 18.32 kg (mean ± SD) were used. Animals were kept out at pasture in pens and supplemented with alfalfa pellets, fresh water *ad libitum*. All males were shorn during the month of November.

### Semen Collection

Semen collections were carried out between the months of April and October using electroejaculation (EE) under general anesthesia according to Director et al. (18). The frequency of collection for each male was determined randomly. As EE requires general anesthesia, this method was not used on the same male at an interval of <15 days and the collection frequency for each male was between 15 and 21 days. All procedures were approved by the Committee for the Use and Care of Laboratory Animals (CICUAL) of the Faculty of Veterinary Sciences of the University of Buenos Aires (protocol 2017/84).

### Llama Sperm Preservation Using Air-Drying

A total of 16 ejaculates from 4 males (*n* = 4, *r* = 4) were processed according to Giuliano et al. (19). Briefly, each sample was diluted 4:1 in a 0.1% collagenase solution (1 mg/ml) in TALP medium. Samples were subsequently incubated 4 min at 37°C and then centrifuged 8 min at 800 *g*. The pellet was re-suspended to a concentration of 20 million sperm/ml in TALP. Then the samples were placed onto sterile slides forming lines and were left to dry under laminar flow for ~15 min. After this, the slides were placed into Falcon centrifuge tubes covered with aluminum foil and were kept at 5°C until evaluation. To carry out the evaluations, TALP medium was placed on the slides and then aspirated with a micropipette and placed in a microcentrifuge tube (Eppendorf® AG, Germany).

### Evaluation of Sperm Characteristics

Seminal characteristics (motility, morphology, membrane function and integrity or viability) were evaluated in raw semen and in air-dried samples preserved at 5°C for 30 min.

Sperm motility was evaluated using a phase contrast microscope (100 x magnification) and a warm stage (37°C). Sperm numbers were calculated using a Neubauer hemocytometer. The hypoosmotic swelling (HOS) test for evaluating membrane function, and the fluorochromes 6-carboxyfluorescein diacetate (CFDA) and propidium iodide (PI) for evaluating membrane integrity or viability, were used according to Giuliano et al. (20). Briefly, for the HOS test, samples were incubated (37°C) in a hypoosmotic solution containing fructose and sodium citrate (50 mOsmol/L). After incubation, a minimum of 200 spermatozoa were evaluated using a phase contrast microscope (400 x magnification). Sperm showing the characteristic swelling of the tail were classified as HOS positive, having a functional plasma membrane. For evaluating membrane integrity or viability, samples were incubated (37°C) with CFDA and PI in an isotonic saline solution described by Harrison and Vickers (21) (NaCl 140 mM, glucose 10 mM, ClK 2.5 mM, polyvinyl-pyrrolidone 0.5 mg/ml and HEPES 20 mM). A minimum of 200 spermatozoa were evaluated per sample using an epifluorescence microscope with a rhodamine and standard fluorescein filter set (400 x magnification). Spermatozoa that fluoresced green throughout their length were classified as being viable (intact plasma membrane) while sperm nuclei that fluoresced red were classified as non-viable (damaged plasma membrane).

Sperm morphology was assessed placing a micro droplet between a slide and coverslip and using a phase contrast microscope (1,000 x magnification). For each sample, 200 spermatozoa were evaluated, and were classified into one of the following categories: normal, abnormal head, detached head, abnormal tail and cytoplasmic droplet. Thus, percentages of spermatozoa with normal or altered morphology were determined.

### Sperm DNA Evaluation

DNA evaluation was carried out in raw semen and in air-dried samples preserved for 30 min and for 7, 14, 21, 30, and 60 days, using two methods.

### Toluidine Blue (TB) Stain

The TB stain was carried out according Carretero et al. (22) to evaluate the degree of condensation/decondensation of llama sperm chromatin. Briefly, smears were made on clean, non-greasy slides and once dry were submerged for 2 min in ethanol 96° to fix them. Then slides were covered with 2 ml of a working solution of TB (0.02%). Preparations were observed directly under immersion oil (1,000 x magnification) evaluating a minimum of 200 spermatozoa per smear. Sperm were classified into three groups according to the degree of chromatin condensation: light blue (negative, condensed chromatin), light violet (intermediate, some degree of decondensation) and dark blue-violet (positive, high degree of decondensation). Positive and intermediate sperm were considered to have altered chromatin condensation. Dithiothreitol (DTT) 1% in distilled water was used as a positive control for sperm DNA decondensation.

### Sperm Chromatin Dispersion Assay (SCD)

The SCD assay was carried out according to Carretero et al. (23) to evaluate the degree of DNA fragmentation. Briefly, aliquots of 50 μl of sperm suspension (5 million sperm/ml in PBS) were mixed with low-melting-point aqueous agarose and pipetted onto a glass slide previously coated with normal-melting-point aqueous agarose, covered with a coverslip and left to solidify at 4°C for 10 min. Then, each slide was incubated in different lysing solutions, dehydrated in sequential ethanol baths and stained with Giemsa. Preparations were observed directly under immersion oil (1,000 x magnification) evaluating a minimum of 200 spermatozoa per sample. Sperm were classified into the following categories: intact DNA (nuclei with large DNA dispersion halos + nuclei with medium-sized halos) and fragmented DNA (nuclei with small halos + nuclei with no halo). Samples incubated with NaOH 0.3 M during 30 min were used as a positive control of sperm DNA fragmentation.

### Statistical Analysis

Statistical analysis was carried out using the InfoStat software. Normal distribution and homogeneity of variances of the data was checked by Shapiro-Wilk Normality test and an ANOVA, respectively. The level of significance was set at 0.05 for all analysis.

A Wilcoxon test was used to compare sperm motility, morphology, plasma membrane function and integrity and a paired Student *t*-test was used to compare chromatin condensation and DNA integrity, between raw semen and samples air-dried for 30 min. A split plot design was used to compare the degree of chromatin condensation between the different periods of preservation of the air-dried samples, blocking the males. Whereas, a Kruskal Wallis test was used to evaluate DNA fragmentation (SCD test) between the air-dried samples.

## Results

### Sperm Characteristics

Air-dried sperm were immotile in all samples. The percentages of sperm with membrane integrity (viable sperm) and with functional membranes (HOS positive) were significantly lower in air-dried samples compared to raw semen (*p* < 0.05). Sperm morphology was not altered due to the process of air-drying (Table 1).

**Table 1:** Seminal characteristics evaluated in raw semen and air-dried llama sperm preserved at 5°C during 30 min.

**Sperm characteristics**	**Raw semen(%)**	**Air-dried sperm (%)**
Motility	22.0, 10.9^a^	0.0^b^
Membrane function (HOS test)	36.8, 13.0^a^	2.2, 1.3^b^
Membrane integrity (viability)	60.1, 24.2^a^	0.2, 0.5^b^
Normal morphology	65.1, 14.3^a^	66.0, 4.7^a^
Abnormal heads	12.2, 2.8^a^	18.0, 2.7^a^
Detached heads	2.0, 1.6^a^	3.0, 0.5^a^
Abnormal tails	7.4, 2.4^a^	10., 6.1^a^
Cytoplasmatic droplets	13.1, 13.9^a^	3.0, 2.4^a^

*Values are expressed as mean ± SD (n = 4; r = 4)*.

a, b *Different letters between columns indicate significant differences for each sperm characteristic evaluated (p < 0.05)*.

### Sperm DNA Condensation

**Figure 1:**
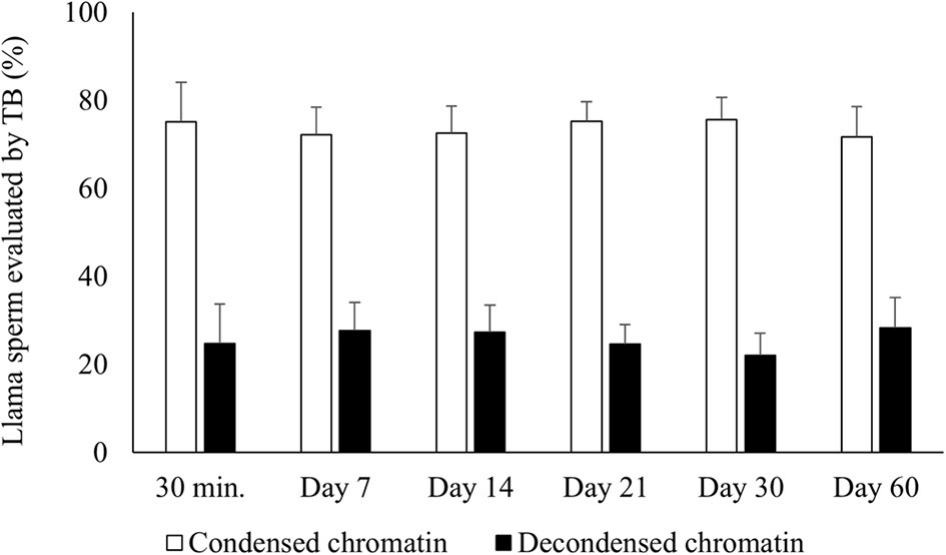
Percentages of air-dried llama sperm with condensed (TB negative) and decondensed chromatin (TB intermediate + positive). Air-drying was evaluated after different times of preservation (30 min and 7, 14, 21, 30, and 60 days) (*n* = 4, *r* = 4). TB, Toluidine blue stain for evaluating sperm chromatin condensation. No significant differences were observed between the periods of preservation within each category (*p* > 0.05).

There was no significant difference for chromatin condensation either between raw semen and samples air-dried for 30 min or between the different periods of preservation of air-dried samples (Figure 1). In addition, 100% of the sperm incubated with DTT (control) were TB positive.

### Sperm DNA Fragmentation

**Figure 2:**
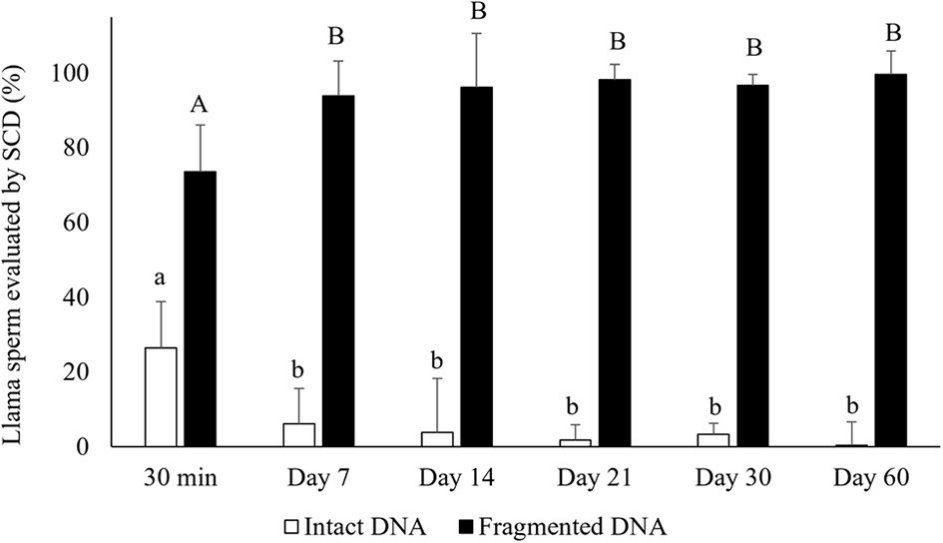
Percentages of sperm with intact DNA (large dispersion halos + medium sized halos) and fragmented DNA (small halos + nuclei with no halo) in air-dried llama sperm samples. The effect of air-drying on sperm was evaluated after different periods of preservation (30 min and 7, 14, 21, 30, and 60 days) (*n* = 4, *r* = 4). SCD, Sperm Chromatin Dispersion test, used for evaluating sperm DNA fragmentation. ^a, b^Different letters indicate significant differences in percentages of intact DNA between periods of preservation (*p* < 0.05). ^A, B^Different letters indicate significant differences in percentages of fragmented DNA between periods of preservation (*p* < 0.05).

Air-drying significantly decreased the percentages of sperm with intact DNA compared to raw semen (intact DNA: 26.4 ± 14.4 and 82.5 ± 12.5% for air-dried sperm for 30 min and raw semen, respectively, mean ± SD). The period of preservation of air-dried samples affected the percentages of sperm with intact DNA, observing a significant decrease from day 7 of preservation (*p* < 0.05) (Figure 2). In addition, 100% of the sperm incubated with NaOH (control) presented fragmented DNA (no halos).

## Discussion

This is the first study to evaluate the simple, inexpensive method of air-drying to preserve llama spermatozoa parameters. Not surprisingly, llama air-dried sperm were immotile. The loss of motility of lyophilized rabbit sperm has been reported (24), as well as that of desiccated rhesus macaque semen submitted to nitrogen gas (25). Sitaula et al. (26) reported that changes in cell volume due to exposition to hyper or hypo-osmotic solutions results in an irreversible loss of motility, showing that one of the main factors that contributes to this loss is an osmotic effect. Similarly, viability or membrane integrity of air-drying llama sperm was very low (0.2 ± 0.5%), coinciding with reports from other authors evaluating freeze-dried mice, rabbit and bull sperm (24, 27, 28). Hence, the osmotic changes that sperm undergo, both during air-drying and when the samples are re-hydrated, could be the cause of the lack of motility and the extremely low percentages of viable sperm observed in this study. However, embryos have been obtained using ICSI with both non-motile and dead sperm in other species (9, 27).

Sperm morphology was not altered by the process of air-drying. Due to the large morphological variability of human spermatozoa (29) the influence of sperm morphology on ICSI results has been extensively studied in men, though with contradictory results (30–33). However, despite similarly variable morphology of llama sperm and the reported success in obtaining llama embryos using ICSI with ejaculated sperm (34, 35), this possible influence has not been studied in SACs. It has been proposed that subtle sperm organellar malformations cannot be detected either by the morphologist at 1,000 x magnification or by the embryologist at 200x−400x magnification. In this context, a new method for the detailed morphological evaluation of motile spermatozoa in real time: the motile sperm organellar morphology examination (MSOME) has been developed, achieving a magnification of 6,300x (36). Using MSOME, human sperm nucleus morphology has positively correlated with fertilization, implantation and pregnancy by ICSI (36, 37). These results further highlight the importance of the sperm nucleus in Assisted Reproductive Techniques (ART), especially ICSI.

With regard to the sperm nucleus, two different aspects were evaluated: the degree of chromatin condensation using TB and the degree of DNA fragmentation using the SCD test. In this study, the process of air-drying did not affect llama chromatin condensation, however, a high percentage of sperm with fragmented DNA was observed in the air-dried samples, as early as 30 min after preservation. This was surprising as we assumed that both aspects (chromatin condensation and DNA integrity) would be similarly affected. Previously, when adapting the SCD test to evaluate llama sperm, we proposed that sperm with fragmented DNA fail to produce a halo because the DNA fragments could be interacting within the llama sperm head by complementary bases (adenine-thymine; guanine-cytosine) making cohesive ends and as a result do not disperse around the nucleus core (23). It is conceivable that despite DNA being fragmented after air-drying, the fragments remained linked at their complementary bases and thus the sperm possibly did not have sites for TB to bind. Additionally, we have previously observed that llama sperm subjected to incubation at 100°C, exposed to UV or incubated with NaOH showed 100% DNA fragmentation in SCD test, but the same samples stained with TB, did not show increased percentages of sperm with decondensed chromatin compared to untreated samples (unpublished data). Those results coincide with what we observed in this study, where despite DNA integrity being significantly decreased (SCD test), chromatin condensation (TB stain) was not affected. If this hypothesis proves to be correct, this could also be an indication that perhaps the SCD test is a more sensitive indicator of DNA alteration than the TB stain.

Although various reports have studied DNA fragmentation in sperm preserved using these alternative methods, to our knowledge there are no reports that evaluate their degree of chromatin condensation. Similar to our results, Klooster et al. (25) desiccated monkey sperm and reported high percentages of sperm with fragmented DNA using the TUNEL technique (93.2–95.5%) in samples preserved during 7 to 10 days at room temperature and at −80°C. Also, Alonso et al. (11) observed that equine sperm DNA fragmentation, evaluated with SCD, increased as the period of air-dried preservation increased (from 32 to 53% in air-dried sperm preserved during 2 days and 4 weeks, respectively). Two possible explanations for the increase in the percentages of sperm with fragmented DNA observed in llama air-dried samples could be a rise in reactive oxygen species (ROS) production and/or the release of endonucleases. The generation of low levels of ROS by spermatozoa plays an important role in different physiological events such as sperm capacitation, acrosome reaction, hyperactivation and sperm-oocyte fusion (38–41). However, high levels of ROS have been associated with cell damage, such as lipid peroxidation and DNA fragmentation (42, 43). Burnaugh et al. (44) incubated equine spermatozoa for 15 min at 38°C under hyperosmotic or hypoosmotic solutions and reported an increase in superoxide production. The changes in osmolarity that occur during the air-drying process could induce an increase in ROS levels and consequently alter sperm DNA. Regarding endonucleases, these sperm enzymes are leaked from plasma membrane-damaged spermatozoa during freeze-drying or freezing without cryoprotectants (27), are activated by divalent cation Ca^2+^ and Mg^2+^ (45) and have been proposed by Nakai et al. (46) to be one the causes of DNA fragmentation in lyophilized (freeze-dried) boar spermatozoa. Taking this into account, Nakai et al. (46) suggested that the use of chelating agents could decrease sperm DNA damage by chelating divalent cations that activate endonucleases. These authors observed that porcine spermatozoa, freeze-dried in the presence of chelating agents such as ethylenediaminetetraacetic acid (EDTA) and ethylene glycoltetraacetic acid (EGTA), presented lower DNA fragmentation values than the control group (4.1 *vs*. 12.2%). Likewise, Sitaula et al. (26) hypothesized that the addition of sugars, antioxidants and chelators to the media could help reduce oxidative stress, thereby minimizing membrane and mitochondrial damage during desiccation. With this in mind and considering the importance of developing a simple method of gamete conservation which would facilitate working with wild species under precarious conditions, it would be interesting to assay the use of media with the addition of sugars, antioxidants and/or chelating agents to evaluate if these substances have a beneficial effect on sperm air-drying and are able to preserve SAC sperm DNA integrity.

## Conclusions

The air-drying process has a negative effect on llama sperm DNA, therefore the media used will need to be improved to protect DNA and be able to implement this technique in these species.

## Data Availability Statement

The raw data supporting the conclusions of this article will be made available by the authors, without undue reservation.

## Ethics Statement

The animal study was reviewed and approved by Committee for the Use and Care of Laboratory Animals (CICUAL) of the Faculty of Veterinary Sciences of the University of Buenos Aires (protocol 2017/84).

## Author Contributions

MIC designed, carried out the study, and wrote the manuscript. MGC, CA, and FF helped collect the samples, critically read, and corrected the manuscript. MG performed the statistical analysis. DN designed and directed the study and critically read and corrected the manuscript. All authors contributed to the article and approved the submitted version.

## Conflict of Interest

The authors declare that the research was conducted in the absence of any commercial or financial relationships that could be construed as a potential conflict of interest.

## Abbreviations

SACs: South American Camelids
SCD: Sperm Chromatin Dispersion assay
TB: Toluidine Blue.
